# Vascular Regulation of Hematopoietic Stem Cell Homeostasis, Regeneration, and Aging

**DOI:** 10.1007/s40778-021-00198-2

**Published:** 2021-09-04

**Authors:** Pradeep Ramalingam, Jason M. Butler, Michael G. Poulos

**Affiliations:** 1 Center for Discovery and Innovation, Hackensack Meridian Health, Nutley, NJ USA; 2 Molecular and Experimental Therapeutic Research in Oncology Program, Georgetown University, Washington, DC USA; 3 Department of Medicine, Division of Regenerative Medicine, Weill Cornell Medical College, New York, NY USA

**Keywords:** Vascular niche, HSC niche, HSC regeneration, Inflammation, Aging, Inflammaging

## Abstract

***Purpose of Review*:**

Hematopoietic stem cells (HSCs) sit at the top of the hierarchy that meets the daily burden of blood production. HSC maintenance relies on extrinsic cues from the bone marrow (BM) microenvironment to balance stem cell self-renewal and cell fate decisions. In this brief review, we will highlight the studies and model systems that define the centralized role of BM vascular endothelium in modulating HSC activity in health and stress.

***Recent Findings*:**

The BM microenvironment is composed of a diverse array of intimately associated vascular and perivascular cell types. Recent dynamic imaging studies, coupled with single-cell RNA sequencing (scRNA-seq) and functional readouts, have advanced our understanding of the HSC-supportive cell types and their cooperative mechanisms that govern stem cell fate during homeostasis, regeneration, and aging. These findings have established complex and discrete vascular microenvironments within the BM that express overlapping and unique paracrine signals that modulate HSC fate.

***Summary*:**

Understanding the spatial and reciprocal HSC-niche interactions and the molecular mechanisms that govern HSC activity in the BM vascular microenvironment will be integral in developing therapies aimed at ameliorating hematological disease and supporting healthy hematopoietic output.

## Introduction

**Fig. 1:**
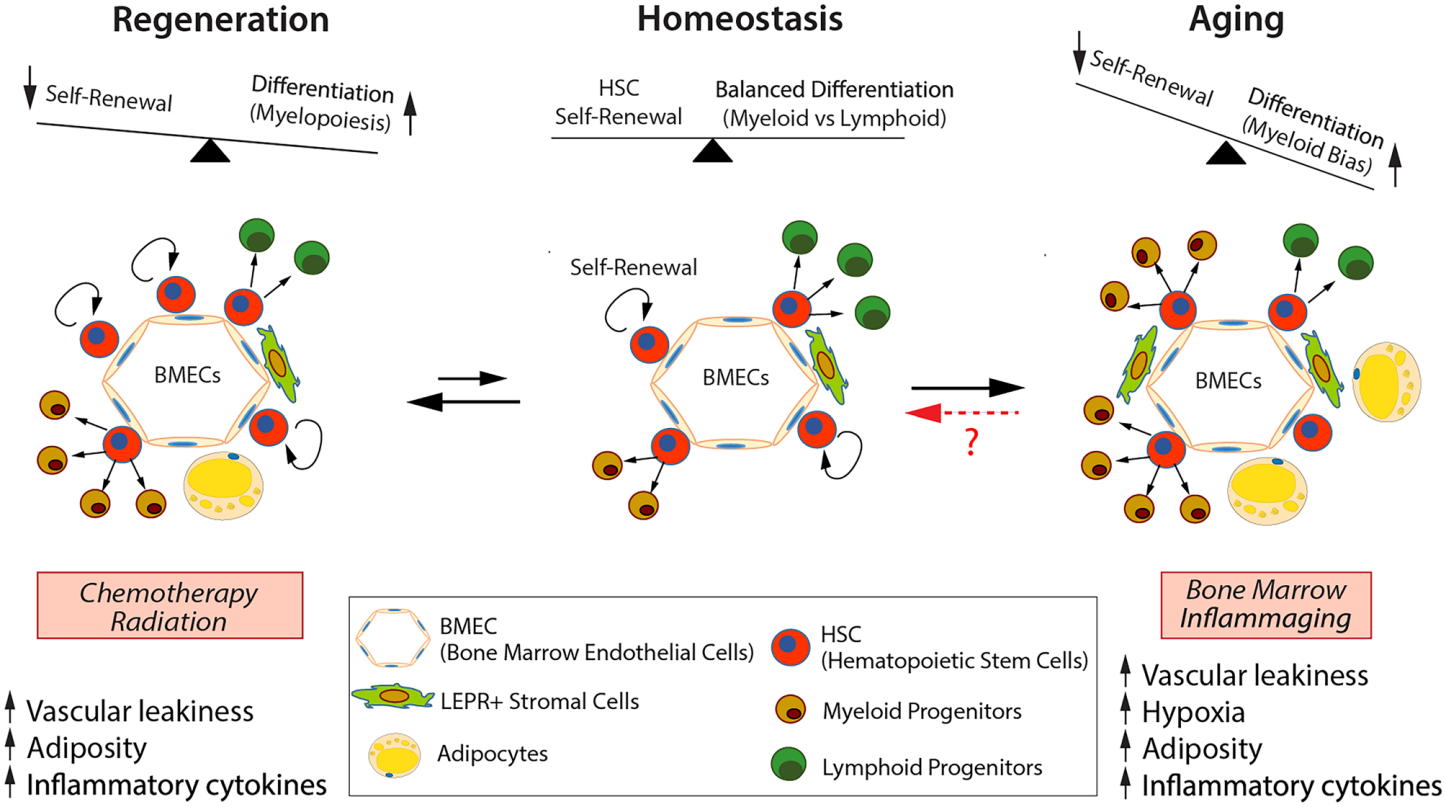
Summary of hematopoietic and vascular changes observed following injury-induced regeneration and during physiological aging. While HSCs return to homeostasis following a myelosuppressive injury, it is not yet known whether aging associated HSC alterations are reversible. BM, bone marrow; EC, endothelial cells; ROS, reactive oxygen species

Endothelial cells (ECs) form the inner lining of the vascular system and serve a diverse array of specialized functions, including delivery of oxygen and nutrients to tissues, regulating platelet adhesion and aggregation, facilitating immune cell trafficking and inflammatory response, and modulating vascular tone [1-3]. BM-derived microvascular endothelial cells also possess specialized hematopoietic-instructive functions that support hematopoietic stem and progenitor cell (HSPC) proliferation and self-renewal directly through membrane-bound or secreted signals [4-8]. Twenty-five years after direct vascular-HSPC interactions were observed in vitro, it has become clear that bone marrow (BM) ECs nucleate an instructive multicellular microenvironment that supports healthy hematopoietic stem cell (HSC) activity in vivo and maintains hematopoietic homeostasis by providing the correct stoichiometry of paracrine factors and optimal metabolic conditions. While vascular niche function in supporting tissue homeostasis is not limited to the BM [9-16], this concise review will focus on our current understanding of the BM endothelial niche, its role in supporting HSC activity, hematopoietic regeneration, and the implications in health and disease (Fig. 1).

## BM Vascular Architecture

Adult BM is a densely vascularized tissue that displays a typical vessel archetype and contains up to 10% of total adult cardiac output at any given time [17, 18]. Blood flows through several high-pressure arteries that enter through cortical bone, branching in a centrifugal pattern into a specialized capillary system (sinusoids), before emptying into the central vein in the diaphysis [19]. Sinusoidal BM endothelium displays a high degree of permeability and a discontinuous basal lamina that allows for the rapid exchange of nutrients and hematopoietic cell trafficking into the peripheral circulation [20, 21]. Oxygen levels in the BM exhibit distinct gradients that are highest in endosteal arteries and lowest near medullary sinusoids [22]. Long bones, including the femur, coxae, and tibia are enriched in red marrow and produce the majority of blood throughout adult life. Emerging live imaging techniques in the marrow space have revealed a dynamic interplay between vascular endothelium and resident-hematopoietic cells [23].

## Distinct Vascular Niches

Tissue-specific vascular beds display specialized endothelial transcriptional profiles that contribute to their development and instructive function [9, 24-26]. In the BM, discrete vascular regions help to spatially define the complex multicellular microenvironments that support hematopoiesis. Sinusoidal endothelium comprises the vast majority of total adult vascular ECs in the BM and can be subclassified based on their anatomical location, vessel diameter, and gradation of immunophenotypic markers [27, 28]. CDH5^+^SCA1^high^ arteriole vasculature merges with transitional sinusoidal vessels (termed Type H) located in the metaphysis and trabecular regions near cortical bone. Transitional CDH5^+^ sinusoidal endothelium expresses high levels of CD31 and EMCN and empty into an extensively branched network of CDH5^+^CD31^low^EMCN^low^ (Type L) sinusoidal capillaries [29, 30]. Arteriole, Type H transitional, and Type L sinusoidal vascular niches are associated with different pericyte components that cooperatively modulate HSC activity as an entity [31]. Perivascular stroma identified as CXCL12-abundant reticular (CAR) cells and Nestin-GFP bright perivascular cells (Nes^peri^) have been reported to localize to BM sinusoids and arterioles, respectively [30]. Specialized microenvironments ultimately support HSC activity via cooperative vascular and perivascular cell types, typified by vascular endothelium and intimately associated LEPR^+^ stromal cells that maintain HSC function through the expression of *Kitl* and *Cxcl12* [32, 33]. It is important to note that LEPR^+^ perivascular stroma largely encompass CXCL12^+^ BM niche cell types [32, 34]. A more comprehensive description of the integral hematopoietic and stromal cell populations, including megakaryocytes, CD169^+^ macrophages, NG2^+^ stroma, and non-myelinating Schwann cells, and accompanying mechanisms that modulate HSC activity in the BM niche, is covered elsewhere [35, 36]. Until now, vascular and perivascular niche cells were primarily defined functionally in vivo using a combination of imaging, genetic knockouts of HSC-supportive factors and cell ablation mouse models. However, new technological approaches harnessing single-cell RNA sequencing (scRNA-seq) allow for unprecedented exploration of the endothelial and stromal cells that populate the BM vascular niche under homeostatic conditions. In the following paragraphs, we will discuss three recent studies that have defined the non-hematopoietic BM compartment in exquisite detail using complementary approaches.

Baryawno et al. sequenced single cells from erythroid and hematopoietic cell-depleted BM to assess total and unbiased stromal populations [37]. Three distinct *Cdh5*- and *Kdr*-expressing endothelial populations were resolved; endothelial subtypes were confirmed using canonical *Flt4* (*Vegfr3*), *Ly6a* (*Sca1*), and *Vwf* gene expression and imaging. Interestingly, pre-adipogenic LEPR^+^ stroma expressing high levels of *Kitl* and *Cxcl12* were mapped along a continuum with osteolineage potential, reinforcing a previous report that LEPR^+^ BM populations are the primary source of adipocytes and bone in adults [38]. Perivascular niche constituents, including NG2^+^ and NES^+^ stromal cells, appeared as distinct populations when compared to LEPR^+^ trajectories. Tikhonova et al. examined bone marrow stromal and endothelial cellular components at a single-cell resolution by crossing constitutively expressing *cre* mice crossed to *Rosa26*^*tdTom*^ floxed reporters, including *Cdh5*^*cre*^ (pan-endothelial), *Lepr*^*cre*^, (mesenchymal stroma), and *Col2.3*^*cre*^ (osteoblasts) [39]. By using a *cre*-expressing reporter system to purify BM niche components for scRNA-seq, identified niche populations closely align with genetic models widely used to explore more functionally defined niche constituents. Two *Cdh5*^*cre*+^ endothelial populations were annotated in a continuous population, including *Ly6a*^*high*^ arterioles and *Stab2*^*high*^ sinusoids. This does not exclude transitional vessels from these populations but rather emphasizes that Type H transitional vessels are a subset of Type L sinusoidal capillaries and occur on a distinct gene expression gradient that is more easily identified using imaging techniques. In agreement with Baryawno et al., the majority of *Lepr*^*cre*^-labeled stroma expressed adipogenic-associated genes (*Lepr*^*adipo*^) along an osteolineage continuum. Interestingly, *Lepr*^*adipo*^ ESM1^+^ stroma were localized to Type L sinusoidal endothelium in the diaphysis, while *Lepr*^*cre*^-labeled osteolineage cells were located near trabecular bone. Baccin et al. examined unbiased and normalized cellular input for scRNA-seq from BM HSPCs, terminally committed hematopoietic cells, and non-hematopoietic stromal BM cell types in combination with laser capture microdissection (LCM) of BM sections and RNA sequencing (RNA-seq) to assign specific cell–cell interactions within discrete microenvironments [40•]. The authors described two endothelial populations, identified as *Ly6a*^*high*^ arterial and *Emcn*^*high*^ sinusoidal BM ECs. In addition, adipo-primed CAR cells (Adipo-CAR) expressing pro-HSC factors were found to be localized to the sinusoidal niche, while osteo-primed CAR cells (Osteo-CAR) were localized to arterial ECs and non-vascular spaces.

Taken together, these studies provide unifying clarity to the continuum of endothelial and stromal cell types that comprise the HSC-supportive BM perivascular microenvironment. Functional data that describes their cooperative cellular interplay will be vital to understanding shared and unique mechanisms of disease onset and progression. These tools will provide significant insights into cooperative multicellular niche interactions for prospective in silico modeling [41] and to explore dynamic changes in response to chemotherapeutic stress [39] and the development of age-related hematological malignancies [37]. Importantly, genetic manipulations of candidate cell types within the compound HSC-supportive perivascular niche are likely to have off-target effects on adjacent niche cells; these factors must be taken into consideration during data interpretation. Taking a more holistic view of the microenvironment may also inform ex vivo three-dimensional organoid development for testing niche-niche interactions and drug discovery [42].

## HSC Spatial Distribution in the BM

Historically, the elaborate surface marker combinations required to enrich for rare populations of HSCs were not amenable for accurately localizing stem cells in the marrow. In 2005, Kiel et al. defined a two-color immunophenotypic system (CD150^+^CD48^−^CD41^−^) that encompassed repopulating HSC activity; using these markers, *bona fide* in vivo HSCs in the marrow space were localized to sinusoidal endothelial niches [43, 44]. This advance in identification propelled a number of studies aimed at refining the exact HSC location and the cooperative cell types that compose the supportive BM-stem cell niche. Using a *Hox5b*^*mCherry*^ reporter, Chen et al. found that greater than 94% of HSCs were directly attached to CDH5^+^ BM vasculature under steady-state conditions [45]. Kunisaki et al. reported that while the majority of HSCs in the marrow were located <20 μm from sinusoidal vessels (67%), only arterioles displayed a statistically significant enrichment of Ki-67^−^ and EdU^+^ label-retaining HSC association [30], suggesting that peri-arteriolar spaces may represent a quiescent HSC niche, while sinusoids support a more proliferative HSC environment. Subsequent deep imaging of femurs sections with α-catulin^GFP+^cKIT^+^ HSCs by Acar et al. allowed for the sampling of a significantly larger population of HSCs in the marrow, noting that approximately 85% of HSCs were in contact with LEPR^+^ cells and <10 μm from sinusoidal endothelium, but remained distant from transitional vessels and arterioles. Notably, α-catulin^GFP+^cKIT^+^ HSC localization to sinusoids was independent of its cycling status [46]. To refine our understanding of HSC localization within the multicellular niche, Kokkaliaris et al. used deep-tissue imaging of up to four BM niche components simultaneously (examining nine cellular components in all) in femurs and sterna using α-catulin^GFP+^cKIT^+^ or *Mds1*^*GFP/*+^; *Flt3*^*cre*^ labeled HSC models. This model system considered niche cell type combinations to define the multicellular microenvironment that cooperatively interacts with HSCs. In adult α-catulin^GFP+^ mice, 84% of HSCs are located within 10 μm of the BM vasculature, while 66% are < 10 μm from both sinusoidal endothelium and CXCL12^+^ stroma [47••]; α-catulin^GFP+^cKIT^+^ and *Mds1*^*GFP/*+^; *Flt3*^*cre*^ HSC localization was equivalent, with primary sinusoid endothelial and CXCL12^+^ stroma niche association and lesser megakaryocyte involvement. Genetic deletion of canonical pro-HSC factors from BM endothelium and intimately associated perivascular LEPR^+^ stromal cells has previously confirmed the functional importance of the compound vascular niche [32, 33, 48, 49]. α-Catulin^GFP+^cKIT^+^ HSC localization between femur and sternum was also indistinguishable. Moreover, dormant and non-dormant HSCs in *SCL*^*tTA*^*; H2B*^*GFP*^ mice demonstrate overlapping sinusoid and CXCL12^+^ stromal association, suggesting that HSC cycling status does not require specialized sub-localization [47••]. Interestingly, potential HSC niches greatly outnumber HSCs within the BM, raising the possibility that HSC-resident sinusoidal niches possess additional unique supportive cellular or paracrine components. Alternatively, stochastically chosen HSC-niche residency may serve to educate endothelial and CXCL12^+^ stromal cellular components to provide a supportive microenvironment. This possibility is observed experimentally by Tamplin et al. using a zebrafish model system with a combination of live imaging and electron microscopy; arrival of an HSPC in its adult niche triggers endothelial remodeling (termed endothelial cuddling) in conjunction with a stromal cell to create a customized pro-hematopoietic anatomical microenvironment [50]. While the relative functional contributions of arteriole and sinusoidal niches in HSC maintenance are still not fully resolved, static snapshots of HSCs in the BM reveal overwhelming association with sinusoidal vascular endothelium and CXCL12^+^ stroma. It is important to note that the methodologies and statistical models used to localize HSCs to their corresponding microenvironmental components are not standardized and remain a source of experimental variability.

Until recently, live imaging studies designed to explore in vivo HSC localization, kinetics, and dynamic niche associations in real time within the BM were limited to post-transplantation homing and hematopoietic reconstitution assays [51-55]. However, these approaches are subject to vascular damage and are likely not indicative of native BM microenvironmental-hematopoietic interactions. Transposon-based barcoding of the native hematopoietic hierarchy has revealed measurable differences in HSC contributions to blood production when compared to previous post-transplantation models [56, 57]. In this domain, Christodoulou et al. developed the *Mds1*^*GFP/*+^; *Flt3*^*cre*^ mouse model that faithfully identifies HSCs by visualization with two-photon LASER scanning microscopy (TPLSM) using a single-color reporter [58•]. Real-time imaging of largely quiescent HSCs in calvarial bone revealed their primarily sinusoidal vessel and endosteal localization with low cellular motility. Treatment of *Mds1*^*GFP/*+^; *Flt3*^*cre*^ mice with either fluorouracil or cyclophosphamide/CSF3 led to an increase in HSC cell cycling and motility. In contrast to these results, Upadhaya et al. used TPLSM live imaging of *Pdzk1ip1*^*creERT2*^; *Rosa26*^*tdTom*^ labeled HSCs and observed significant cellular motility in both tibia and calvaria marrow [59]. Dynamic HSC movement was noted in *Fgd5*^*ZsGreen*+^ perisinusoidal spaces with consistent and transient *Kitl*^*GFP*+^ cell interactions. Interestingly, pharmacological inhibition of CXCR4 and integrin signaling disrupted HSC motility. Differences in these observations may be attributed to the number of HSCs sampled, genetic models used to label HSCs, or administration of low doses of tamoxifen. In addition, the *Mds1*^*GFP/*+^; *Flt3*^*cre*^ HSCs sampled represent a subset of total repopulating HSCs and may not reflect the population dynamics observed in *Pdzk1ip1*^*creERT2*^; *Rosa26*^*tdTom*^ HSCs. Nonetheless, both approaches offer significant advances towards understanding direct HSC interactions and behavior in their native microenvironments.

## Endothelial Paracrine Factors Regulating the HSC Niche

To maintain their capacity for balanced self-renewal and differentiation, HSCs receive a diverse array of secreted and membrane-bound paracrine factors from the perivascular niche that can be broadly classified into the following categories: (1) factors promoting HSC quiescence and self-renewal, including KITL and TPO and (2) factors that induce lineage commitment and differentiation into multipotent progenitors (MPPs), including IL1 β and IFNγ [60-63]. The hypothesis for a vascular niche for HSCs was first proposed by Rafii et al. in 1997, based on their observations that ECs lining the BM microvasculature were capable of supporting long-term expansion of human HSPCs ex vivo through their ability to express pro-hematopoietic growth factors, including CSF2, CSF3, and KITL [5, 64]. These elegant studies paved the way for the functional interrogation of BM EC niche cells and their contributions to HSC regulation and hematopoiesis in vivo. Sugiyama et al. demonstrated that conditional deletion of *Cxcl12*, a factor abundantly expressed in perivascular stromal cells and ECs, resulted in severe depletion of HSCs and impaired recovery following myelotoxic injury [65]. By utilizing endothelial-specific mouse models, Butler et al. demonstrated that expression of NOTCH ligands by ECs is essential for HSC self-renewal and BM repopulation [66], while Kobayashi et al. showed that AKT signaling within adult murine ECs promote HSC self-renewal and expansion [6]. These discoveries were followed by a series of studies that identified the roles of various HSC-regulatory paracrine factors expressed by the vascular niche including *Sele* [67], *Kitl* [32], *Cxcl12* [33, 68], *Jag1* [49], *Egf* [69], and *Ptn* [70]. In addition to this core group of EC-derived factors in HSC maintenance, modulation of fundamental signaling networks within the BM vascular endothelium also plays a central role in HSC homeostasis. For instance, the inhibition of mTOR signaling in young mice, either by rapamycin treatment or EC-specific genetic deletion, results in premature aging phenotypes in young HSCs [71], whereas constitutive suppression of endothelial NF-κB signaling enhances HSC activity during homeostasis [72]. Following the recognition of the adult BM vasculature as a functional niche for HSCs, it was soon discovered that the stromal cells sharing an intimate relationship with the vasculature also played a significant role in HSC maintenance. As discussed, the identification of *Kitl* and *Cxcl12* as key regulators of HSC function spurred the search for other stromal cells within the BM that express these factors, leading to the identification of CAR cells [73], NES^+^ cells [74], non-myelinating Schwann cells [30], NG2^+^ cells [30], and LEPR^+^ cells [38]. Collectively, these studies demonstrated that BM ECs serve as a scaffold for the assembly of an array of perivascular stromal cells to cooperatively establish a compound vascular niche that supports HSC maintenance during homeostasis and augments hematopoietic recovery following myelosuppressive injury.

## BM Vascular Regeneration

Vascular regeneration following myeloablative injury is a keystone event in reestablishing hematopoiesis in the marrow. In response to irradiation, BM endothelial gene expression is rapidly altered [75-77••]. Sinusoidal endothelium undergoes cell death in a dose-dependent manner in response to ionizing radiation and chemotherapy [78, 79]. Regeneration of FLT4^+^ sinusoidal endothelium in an endothelial-specific *Kdr*^*−/−*^ model is a prerequisite for hematopoietic engraftment and reconstitution [78], while exogenous administration of recombinant VEGFC promotes vascular niche recovery [80]. In response to irradiation, BM sinusoids are regenerated from existing capillaries [79], with contributions from transitional APLN^+^ ECs [77••]. Interestingly, significant defects including capillary dilation and leakiness are observed in the hematopoietic-supportive BM and spleen vasculature following irradiation or treatment with fluorouracil, but ECs are notably less damaged in non-hematopoietic tissues, including the heart, retina, and skin, suggesting a dynamic interplay between loss of hematopoietic cells in response to myeloablation and degree of vascular injury and recovery [77••]. Ablation of pan-hematopoietic cells in the absence of additional vascular injury using a *Vav1*^*cre*^; *Rosa26*^*DTR*^ model also reveals disruption of BM sinusoidal endothelium, suggesting a reciprocal interplay between the BM vasculature and hematopoietic cells [77••]. Interestingly, this creates a therapeutic paradox, as preconditioning regimens precluding hematopoietic stem cell transplantation (HSCT) significantly damage the BM sinusoidal niche required for hematopoietic reconstitution. Following irradiation, localization of donor-derived hematopoietic progenitors to sinusoids provided early evidence that HSPCs may play an important role in host vascular repair [81]. VEGFA-induced *Tie2* expression is upregulated in BM sinusoidal endothelium following radiation or chemotherapy injury, promoting vessel repair [82]. *Angpt1* expression in HSPCs and BM LEPR^+^ mesenchymal stromal cells following irradiation reduces vascular leakiness at the expense of rapid vascular and hematopoietic regeneration [83], potentially acting through NF-kB inhibition [84] and NOTCH activation [85], highlighting the ANGPT1/TIE2 signaling axis as an intriguing biological trade-off that prioritizes vascular integrity over more immediate repair. Reciprocal hematopoietic crosstalk following vascular injury is not limited to stem and progenitor cells. Adoptive transfer of BM-derived granulocytes also direct rapid hematopoietic recovery; BM-resident GR1^+^CD115^−^ granulocytes express *Tnfa* and localize to sinusoidal BM ECs, driving vessel regeneration through endothelial *Tnfr1*/*Tnfr2* signaling and, in turn, hematopoietic reconstitution [86]. Genetic ablation of granulocytes in mice transplanted with *Mrp8*^*cre*^; *iDTR* BM impaired both vascular and hematopoietic recovery in lethally irradiated recipients. Molecular or cellular interventions designed to support vascular integrity following injury may provide a therapeutic window to support hematopoietic engraftment and recovery.

Transplantation of healthy endothelium can also mitigate BM niche damage following injury. Intravenous (IV) administration of ECs derived from non-hematopoietic sources after irradiation improves overall survival and promotes hematopoietic recovery in the absence of exogenous endothelial engraftment [87-89]. Notably, infusion of BM-derived ECs is more effective in mitigating radiation damage to the niche when compared with ECs derived from non-hematopoietic tissues, while more efficiently protecting the HSC pool and inducing more rapid peripheral hematopoietic recovery [8]. Infusion of BM-derived extracellular vesicles (EVs) following radiation phenocopies BM EC-mediated hematopoietic recovery, in part through TIMP1-mediated mitigation of HSPC cell death [90]. The mechanisms that drive hematopoietic recovery following EC/EV transplantation are not fully established, although injury-mitigation likely acts through endothelial delivery of HSC-supportive factors, including PTN, EGF, and NOTCH ligands [49, 66, 69, 70, 91, 92]. In addition, evidence from Doan et al. demonstrates that inhibition of BM EC apoptosis in *Tie2*^*cre*^; *Bak1*^*−/−*^; *Bax*^*fl/−*^ mice promotes overall survival in response to lethal irradiation [93], while Type H transitional and Type L capillary sinusoids are dependent on *Id1*/*Id3* expression for survival and proliferation following sublethal irradiation [94]. Therefore, stabilizing BM sinusoidal endothelium prior to myeloablation may be useful in protecting the vascular niche from cytotoxic insult. Suppression of NF-κB-dependent vascular inflammation also radioprotects the BM microenvironment, including sinusoidal ECs and LEPR^+^ cells [72]. Understanding the mechanisms that protect BM endothelium following injury offers an opportunity to improve existing HSCT outcomes and mitigate myelosuppression following chemotherapy.

## Vascular Inflammation and Aging

Aging leads to a gradual decline of HSC activity and dysregulation of hematopoiesis. The hallmarks of aged HSCs include impaired self-renewal potential, myeloid-biased differentiation, loss of cell polarity, accumulation of DNA damage, and an impaired ability for homing and engraftment [95-102]. Clinically, aging is associated with an increased risk of negative outcomes and treatment failures because elderly patients respond poorly to conditioning strategies necessary for successful HSCT and develop prolonged cytopenias following myelosuppressive therapies used to treat hematological malignancies and other cancers [103]. Accumulating evidence suggests that inflammation plays a dominant role in the HSC aging process [104-107]. Sustained inflammation has been proposed as a key driver of aging-associated hematopoietic defects, including the loss of HSC self-renewal, myeloid-biased differentiation, and a predisposition towards leukemia [104-109]. Studies indicate that even in the absence of active infections, aging is associated with chronic low-grade inflammation that causes myeloid-skewing and detrimental effects on HSCs [104]. This phenomenon has been coined “inflammation-associated aging” or “inflammaging” [110]. Furthermore, inflammation is a crucial component contributing towards increased levels of reactive oxygen species (ROS) known to be detrimental to HSC function [111-113]; in turn, excessive ROS can activate inflammatory signaling, perpetuating HSC dysfunction [114]. Elevated levels of ROS have also been shown to impair HSC engraftment potential and promote myeloid-biased differentiation via activation of the mTOR and MAPK pathways [115, 116]. Collectively, these findings indicate that chronic inflammation likely drives aging-associated HSC defects. However, the signals that initiate and sustain these alterations within the BM remain poorly understood. Given that vascular aging has emerged as the quintessential common denominator in driving age-associated functional decline of every organ system, it is likely that aging of the BM vascular niche plays a crucial role in modulating HSC aging phenotypes [117-120]. Indeed, it has been proposed that inflammation within the BM microenvironment may play key roles in inducing aging-associated alterations within the vascular niche, leading to age-related HSC defects and a predisposition towards myeloid-borne neoplasms [106, 107, 121, 122]. A few studies have explored this idea and investigated aging related alterations in the BM microenvironment [121]. Aging is associated with an increased vascular density in the BM, with a shortening of arteriolar segments and a decrease in arteriole and transitional sinusoidal vessels [123, 124••]. Aging is also associated with increased vascular leakiness, increased hypoxia, and elevated ROS levels within the BM vascular niche, features that are consistent with an inflammatory stress [125]. Sustained endothelial inflammation has been implicated in the initiation of myeloproliferative disorders through the expression of *Csf3* and *Tnf* [126]. Additionally, ECs play a key role in sustaining chronic inflammation [9, 127] and have emerged as an important source of niche-derived inflammatory signals within the BM, including IL1 and CSF3 [108, 128]. We recently demonstrated that chronic vascular inflammation is sufficient to promote premature HSC aging phenotypes, including the loss of self-renewal and skewing towards a myeloid-biased output [129••]. Notably, these HSC aging phenotypes could be reversed by genetic or pharmacological suppression of vascular inflammation, highlighting the potential for rejuvenation of the aged HSC pool by targeting inflammation in the vascular niche [129••]. These studies highlight the possibility of targeting niche-derived inflammation as an approach to mitigate HSC aging phenotypes and improve overall health span [71, 129••-132].

Apart from the insights gained from these studies, our current knowledge on aging-associated functional defects within the BM vascular niche remains limited. Perhaps, the most dramatic characteristic of an aged BM microenvironment is adipocyte infiltration that has been shown to have a negative impact on HSC function [133-135]. The accumulation of adipocytes is indicative of metabolic dysfunction within the aged BM and likely results from increased levels of free fatty acids. In support of this, a recent study reporting an aging-associated increase of free fatty acids within the murine BM, along with a decrease in acylcarnitine levels, suggests impaired beta oxidation [136]. Given that HSC cell fate decisions are intricately intertwined with their metabolic status [137], the phenotypic defects observed in aged HSCs may arise from an altered metabolic microenvironment in the aged BM. Notably, increased levels of free fatty acids have also been shown to activate inflammatory responses and increase oxidative stress, both of which are detrimental to EC health and HSC activity [138]. Given that ECs act as critical gatekeepers of tissue-specific metabolite flux, including free fatty acids, it is possible that aging-induced alterations within the BM vascular endothelium result in metabolic maladaptation and inflammatory stress within the BM microenvironment, leading to HSC defects. Collectively, these findings highlight the critical need for characterization of the physiological perturbations and metabolic alterations of the aged BM vascular niche to obtain molecular insights into aging-associated niche defects, to ultimately guide the development of therapies for rejuvenation.

## Concluding Statement

As our understanding of the individual cell types and factors that comprise the HSC-supportive BM vascular niche comes into focus, new tools and approaches are needed to experimentally validate the mechanisms that govern both HSC activity and cell-to-cell interactions between cooperative niche constituents. Guided by new transcriptional data, single-cell resolution, and the continued identification of paracrine factors that modulate HSC fate decisions, additional high fidelity and inducible *cre*/*lox* and *dre*/*rox* systems will be necessary to interrogate the discrete cells types that cooperatively dictate vascular and perivascular adult-specific HSC niche function. The development of three-dimensional in silico and ex vivo models of the in vivo BM niche will limit confounding cell-to-cell cross talk and bystander effects between the vasculature, perivascular, and hematopoietic cell types and provide a more direct and rapid platform for drug discovery. Strategies that target BM vasculature to preserve homeostatic instructive function during stress hematopoiesis and aging provide an exciting therapeutic avenue to mitigate HSC damage and improve patient outcomes.

## References

[1] Sang Y, et al. Interplay between platelets and coagulation*.* Blood Rev. 2020;100733. 10.1016/j.blre.2020.100733 PMC7354275 32682574

[2] Muller WA (2016). Transendothelial migration: unifying principles from the endothelial perspective. Immunol Rev.

[3] Kruger-Genge A, et al. Vascular endothelial cell biology: an update*.* Int J Mol Sci. 2019;20(18). 10.3390/ijms20184411 PMC6769656 31500313

[4] Rafii S (1994). Isolation and characterization of human bone marrow microvascular endothelial cells: hematopoietic progenitor cell adhesion. Blood.

[5] Rafii S (1995). Human bone marrow microvascular endothelial cells support long-term proliferation and differentiation of myeloid and megakaryocytic progenitors. Blood.

[6] Kobayashi H (2010). Angiocrine factors from Akt-activated endothelial cells balance self-renewal and differentiation of haematopoietic stem cells. Nat Cell Biol.

[7] Butler JM (2012). Development of a vascular niche platform for expansion of repopulating human cord blood stem and progenitor cells. Blood.

[8] Poulos MG (2015). Vascular platform to define hematopoietic stem cell factors and enhance regenerative hematopoiesis. Stem Cell Reports.

[9] Rafii S, Butler JM, Ding BS (2016). Angiocrine functions of organ-specific endothelial cells. Nature.

[10] Bhang DH (2018). Testicular endothelial cells are a critical population in the germline stem cell niche. Nat Commun.

[11] Cao Z (2016). Targeting of the pulmonary capillary vascular niche promotes lung alveolar repair and ameliorates fibrosis. Nat Med.

[12] Ding BS (2014). Divergent angiocrine signals from vascular niche balance liver regeneration and fibrosis. Nature.

[13] Ding BS (2010). Inductive angiocrine signals from sinusoidal endothelium are required for liver regeneration. Nature.

[14] Oh M (2020). Endothelial-initiated crosstalk regulates dental pulp stem cell self-renewal. J Dent Res.

[15] Shen Q (2004). Endothelial cells stimulate self-renewal and expand neurogenesis of neural stem cells. Science.

[16] Tavazoie M (2008). A specialized vascular niche for adult neural stem cells. Cell Stem Cell.

[17] Tomlinson RE, Silva MJ (2013). Skeletal blood flow in bone repair and maintenance. Bone Res.

[18] Ramasamy SK (2017). Structure and functions of blood vessels and vascular niches in bone. Stem Cells Int.

[19] Marenzana M, Arnett TR (2013). The key role of the blood supply to bone. Bone Res.

[20] Inoue S, Osmond DG (2001). Basement membrane of mouse bone marrow sinusoids shows distinctive structure and proteoglycan composition: a high resolution ultrastructural study. Anat Rec.

[21] Itkin T (2016). Distinct bone marrow blood vessels differentially regulate haematopoiesis. Nature.

[22] Spencer JA (2014). Direct measurement of local oxygen concentration in the bone marrow of live animals. Nature.

[23] Reismann D (2017). Longitudinal intravital imaging of the femoral bone marrow reveals plasticity within marrow vasculature. Nat Commun.

[24] Nolan DJ (2013). Molecular signatures of tissue-specific microvascular endothelial cell heterogeneity in organ maintenance and regeneration. Dev Cell.

[25] Ramasamy SK (2016). Regulation of hematopoiesis and osteogenesis by blood vessel-derived signals. Annu Rev Cell Dev Biol.

[26] Kenswil KJG (2018). Characterization of endothelial cells associated with hematopoietic niche formation in humans identifies IL-33 as an anabolic factor. Cell Rep.

[27] Taylor AM, Bordoni B. Histology, blood vascular system, in StatPearls. 2020: Treasure Island (FL). 31985998

[28] Watson EC, Adams RH. Biology of bone: the vasculature of the skeletal system*.* Cold Spring Harb Perspect Med. 2018;8(7). 10.1101/cshperspect.a031559 PMC6027931 28893838

[29] Kusumbe AP, Ramasamy SK, Adams RH (2014). Coupling of angiogenesis and osteogenesis by a specific vessel subtype in bone. Nature.

[30] Kunisaki Y (2013). Arteriolar niches maintain haematopoietic stem cell quiescence. Nature.

[31] Lucas D (2019). Leukocyte trafficking and regulation of murine hematopoietic stem cells and their niches. Front Immunol.

[32] Ding L (2012). Endothelial and perivascular cells maintain haematopoietic stem cells. Nature.

[33] Ding L, Morrison SJ (2013). Haematopoietic stem cells and early lymphoid progenitors occupy distinct bone marrow niches. Nature.

[34] Coutu DL (2017). Three-dimensional map of nonhematopoietic bone and bone-marrow cells and molecules. Nat Biotechnol.

[35] Kokkaliaris KD (2020). Dissecting the spatial bone marrow microenvironment of hematopoietic stem cells. Curr Opin Oncol.

[36] May M, Slaughter A, Lucas D (2018). Dynamic regulation of hematopoietic stem cells by bone marrow niches. Curr Stem Cell Rep.

[37] Baryawno N, et al. A cellular taxonomy of the bone marrow stroma in homeostasis and leukemia*.* Cell. 2019;177(7):1915–1932 e16. 10.1016/j.cell.2019.04.040 PMC6570562 31130381

[38] Zhou BO (2014). Leptin-receptor-expressing mesenchymal stromal cells represent the main source of bone formed by adult bone marrow. Cell Stem Cell.

[39] Tikhonova AN (2019). The bone marrow microenvironment at single-cell resolution. Nature.

[40] • Baccin C, et al. Combined single-cell and spatial transcriptomics reveal the molecular, cellular and spatial bone marrow niche organization*.* Nat Cell Biol. 2020;22(1):38–48. **This study utilizes a combination of single-cell and targeted transcriptional analysis to prospectively map the perivascular BM-HSC niche.** 10.1038/s41556-019-0439-6 PMC7610809 31871321

[41] Desterke C, et al. Inferring gene networks in bone marrow hematopoietic stem cell-supporting stromal niche populations*.* iScience. 2020;23(6):101222. 10.1016/j.isci.2020.101222 PMC7300160 32535025

[42] Ham J, et al. In vitro 3D cultures to reproduce the bone marrow niche*.* JBMR Plus. 2019;3(10):e10228. 10.1002/jbm4.10228 PMC6820578 31687654

[43] Kiel MJ (2005). SLAM family receptors distinguish hematopoietic stem and progenitor cells and reveal endothelial niches for stem cells. Cell.

[44] Yilmaz OH, Kiel MJ, Morrison SJ (2006). SLAM family markers are conserved among hematopoietic stem cells from old and reconstituted mice and markedly increase their purity. Blood.

[45] Chen JY (2016). Hoxb5 marks long-term haematopoietic stem cells and reveals a homogenous perivascular niche. Nature.

[46] Acar M (2015). Deep imaging of bone marrow shows non-dividing stem cells are mainly perisinusoidal. Nature.

[47] •• Kokkaliaris KD, et al. Adult blood stem cell localization reflects the abundance of reported bone marrow niche cell types and their combinations. Blood. 2020;136(20):2296–2307. **This study comprehensively quantifies HSC-microenvironmental associations by deep imaging of HSCs and multiple niche components simultaneously.** 10.1182/blood.2020006574 PMC8209553 32766876

[48] Asada N (2017). Differential cytokine contributions of perivascular haematopoietic stem cell niches. Nat Cell Biol.

[49] Poulos MG (2013). Endothelial jagged-1 is necessary for homeostatic and regenerative hematopoiesis. Cell Rep.

[50] Tamplin OJ (2015). Hematopoietic stem cell arrival triggers dynamic remodeling of the perivascular niche. Cell.

[51] Lo Celso C (2009). Live-animal tracking of individual haematopoietic stem/progenitor cells in their niche. Nature.

[52] Celso Lo, Lin CP, Scadden DT. In vivo imaging of transplanted hematopoietic stem and progenitor cells in mouse calvarium bone marrow. Nat Protoc. 2011;6(1):1–14. 10.1038/nprot.2010.168 PMC3382040 21212779

[53] Kim S (2017). Extended time-lapse in vivo imaging of tibia bone marrow to visualize dynamic hematopoietic stem cell engraftment. Leukemia.

[54] Bixel MG (2017). Flow dynamics and HSPC homing in bone marrow microvessels. Cell Rep.

[55] Busch K, Rodewald HR. Unperturbed vs. post-transplantation hematopoiesis: both in vivo but different. Curr Opin Hematol. 2016;23(4):295–303. 10.1097/MOH.0000000000000250 PMC4900429 27213498

[56] Sun J (2014). Clonal dynamics of native haematopoiesis. Nature.

[57] Rodriguez-Fraticelli AE (2018). Clonal analysis of lineage fate in native haematopoiesis. Nature.

[58] • Christodoulou C, et al. Live-animal imaging of native haematopoietic stem and progenitor cells*.* Nature. 2020;578(7794):278–283. **This study develops a simplified genetic system to image HSCs in their native microenvironment in real time.** 10.1038/s41586-020-1971-z PMC7021587 32025033

[59] Upadhaya S, et al. Intravital imaging reveals motility of adult hematopoietic stem cells in the bone marrow Niche*.* Cell Stem Cell. 2020. 10.1016/j.stem.2020.06.003 PMC7415613 32589864

[60] Seita J, Weissman IL (2010). Hematopoietic stem cell: self-renewal versus differentiation. Wiley Interdiscip Rev Syst Biol Med.

[61] Li J (2011). Quiescence regulators for hematopoietic stem cell. Exp Hematol.

[62] Robb L (2007). Cytokine receptors and hematopoietic differentiation. Oncogene.

[63] King KY, Goodell MA (2011). Inflammatory modulation of HSCs: viewing the HSC as a foundation for the immune response. Nat Rev Immunol.

[64] Rafii S (1997). Regulation of hematopoiesis by microvascular endothelium. Leuk Lymphoma.

[65] Sugiyama T (2006). Maintenance of the hematopoietic stem cell pool by CXCL12-CXCR4 chemokine signaling in bone marrow stromal cell niches. Immunity.

[66] Butler JM (2010). Endothelial cells are essential for the self-renewal and repopulation of Notch-dependent hematopoietic stem cells. Cell Stem Cell.

[67] Winkler IG (2012). Vascular niche E-selectin regulates hematopoietic stem cell dormancy, self-renewal and chemoresistance. Nat Med.

[68] Greenbaum A (2013). CXCL12 in early mesenchymal progenitors is required for haematopoietic stem-cell maintenance. Nature.

[69] Doan PL (2013). Epidermal growth factor regulates hematopoietic regeneration after radiation injury. Nat Med.

[70] Himburg HA (2012). Pleiotrophin regulates the retention and self-renewal of hematopoietic stem cells in the bone marrow vascular niche. Cell Rep.

[71] Ramalingam P, et al. Endothelial mTOR maintains hematopoiesis during aging*.* J Exp Med. 2020;217(6). 10.1084/jem.20191212 PMC7971143 32289154

[72] Poulos MG (2016). Endothelial-specific inhibition of NF-kappaB enhances functional haematopoiesis. Nat Commun.

[73] Omatsu Y (2010). The essential functions of adipo-osteogenic progenitors as the hematopoietic stem and progenitor cell niche. Immunity.

[74] Mendez-Ferrer S (2010). Mesenchymal and haematopoietic stem cells form a unique bone marrow niche. Nature.

[75] Himburg HA (2016). A molecular profile of the endothelial cell response to ionizing radiation. Radiat Res.

[76] Cary L, et al. Bone marrow endothelial cells influence function and phenotype of hematopoietic stem and progenitor cells after mixed neutron/gamma radiation. Int J Mol Sci. 2019;20(7). 10.3390/ijms20071795 PMC6480930 30978983

[77] •• Chen Q, et al. Apelin(+) Endothelial niche cells control hematopoiesis and mediate vascular regeneration after myeloablative injury. Cell Stem Cell. 2019;25(6):768–783 e6. **This study describes a novel regenerative BM endothelial cell that mediates vascular recovery following injury.** 10.1016/j.stem.2019.10.006 PMC6900750 31761723

[78] Hooper AT (2009). Engraftment and reconstitution of hematopoiesis is dependent on VEGFR2-mediated regeneration of sinusoidal endothelial cells. Cell Stem Cell.

[79] Li XM (2008). Bone marrow sinusoidal endothelial cells undergo nonapoptotic cell death and are replaced by proliferating sinusoidal cells in situ to maintain the vascular niche following lethal irradiation. Exp Hematol.

[80] Fang S (2020). VEGF-C protects the integrity of the bone marrow perivascular niche in mice. Blood.

[81] Slayton WB (2007). The role of the donor in the repair of the marrow vascular niche following hematopoietic stem cell transplant. Stem Cells.

[82] Kopp HG (2005). Tie2 activation contributes to hemangiogenic regeneration after myelosuppression. Blood.

[83] Zhou BO, L Ding, SJ Morrison. Hematopoietic stem and progenitor cells regulate the regeneration of their niche by secreting Angiopoietin-1*.* Elife. 2015;4:e05521. 10.7554/eLife.05521 PMC4411515 25821987

[84] Hughes DP, Marron MB, Brindle NP (2003). The antiinflammatory endothelial tyrosine kinase Tie2 interacts with a novel nuclear factor-kappaB inhibitor ABIN-2. Circ Res.

[85] Shao L (2019). A Tie2-Notch1 signaling axis regulates regeneration of the endothelial bone marrow niche. Haematologica.

[86] Bowers E (2018). Granulocyte-derived TNFalpha promotes vascular and hematopoietic regeneration in the bone marrow. Nat Med.

[87] Chute JP (2007). Transplantation of vascular endothelial cells mediates the hematopoietic recovery and survival of lethally irradiated mice. Blood.

[88] Salter AB (2009). Endothelial progenitor cell infusion induces hematopoietic stem cell reconstitution in vivo. Blood.

[89] Li B (2010). Endothelial cells mediate the regeneration of hematopoietic stem cells. Stem Cell Res.

[90] Piryani SO (2019). Endothelial cell-derived extracellular vesicles mitigate radiation-induced hematopoietic injury. Int J Radiat Oncol Biol Phys.

[91] Guo P (2017). Endothelial jagged-2 sustains hematopoietic stem and progenitor reconstitution after myelosuppression. J Clin Invest.

[92] Himburg HA (2010). Pleiotrophin regulates the expansion and regeneration of hematopoietic stem cells. Nat Med.

[93] Doan PL (2013). Tie2(+) bone marrow endothelial cells regulate hematopoietic stem cell regeneration following radiation injury. Stem Cells.

[94] Gadomski S, et al. Id1 and Id3 maintain steady-state hematopoiesis by promoting sinusoidal endothelial cell survival and regeneration*.* Cell Rep. 2020;31(4):107572. 10.1016/j.celrep.2020.107572 PMC8459380 32348770

[95] Cho RH, Sieburg HB, Muller-Sieburg CE (2008). A new mechanism for the aging of hematopoietic stem cells: aging changes the clonal composition of the stem cell compartment but not individual stem cells. Blood.

[96] Dykstra B, de Haan G (2008). Hematopoietic stem cell aging and self-renewal. Cell Tissue Res.

[97] Rossi DJ (2005). Cell intrinsic alterations underlie hematopoietic stem cell aging. Proc Natl Acad Sci U S A.

[98] Van Zant G, Liang Y (2003). The role of stem cells in aging. Exp Hematol.

[99] Chambers SM, et al. Aging hematopoietic stem cells decline in function and exhibit epigenetic dysregulation*.* PLoS Biol, 2007;5(8):e201. 10.1371/journal.pbio.0050201 PMC1925137 17676974

[100] Pang WW (2011). Human bone marrow hematopoietic stem cells are increased in frequency and myeloid-biased with age. Proc Natl Acad Sci U S A.

[101] Geiger H, de Haan G, Florian MC (2013). The ageing haematopoietic stem cell compartment. Nat Rev Immunol.

[102] Kowalczyk MS (2015). Single-cell RNA-seq reveals changes in cell cycle and differentiation programs upon aging of hematopoietic stem cells. Genome Res.

[103] Balducci L (2003). Myelosuppression and its consequences in elderly patients with cancer. Oncology (Williston Park).

[104] Pietras EM (2017). Inflammation: a key regulator of hematopoietic stem cell fate in health and disease. Blood.

[105] Schuettpelz LG, Link DC (2013). Regulation of hematopoietic stem cell activity by inflammation. Front Immunol.

[106] Kovtonyuk LV (2016). Inflamm-aging of hematopoiesis, hematopoietic stem cells, and the bone marrow microenvironment. Front Immunol.

[107] Leimkuhler NB, Schneider RK (2019). Inflammatory bone marrow microenvironment. Hematology Am Soc Hematol Educ Program.

[108] Pietras EM (2016). Chronic interleukin-1 exposure drives haematopoietic stem cells towards precocious myeloid differentiation at the expense of self-renewal. Nat Cell Biol.

[109] Lussana F, Rambaldi A (2017). Inflammation and myeloproliferative neoplasms. J Autoimmun.

[110] Franceschi C, Campisi J (2014). Chronic inflammation (inflammaging) and its potential contribution to age-associated diseases. J Gerontol A Biol Sci Med Sci.

[111] Zuo L, et al. Inflammaging and oxidative stress in human diseases: from molecular mechanisms to novel treatments. Int J Mol Sci. 2019;20(18). 10.3390/ijms20184472 PMC6769561 31510091

[112] Urao N, Ushio-Fukai M (2013). Redox regulation of stem/progenitor cells and bone marrow niche. Free Radic Biol Med.

[113] Liang R, Ghaffari S (2014). Stem cells, redox signaling, and stem cell aging. Antioxid Redox Signal.

[114] Forrester SJ (2018). Reactive oxygen species in metabolic and inflammatory signaling. Circ Res.

[115] Jang YY, Sharkis SJ (2007). A low level of reactive oxygen species selects for primitive hematopoietic stem cells that may reside in the low-oxygenic niche. Blood.

[116] Ito K (2006). Reactive oxygen species act through p38 MAPK to limit the lifespan of hematopoietic stem cells. Nat Med.

[117] El Assar M (2012). Mechanisms involved in the aging-induced vascular dysfunction. Front Physiol.

[118] El Assar M, Angulo J, Rodriguez-Manas L (2013). Oxidative stress and vascular inflammation in aging. Free Radic Biol Med.

[119] Ungvari Z (2018). Mechanisms of vascular aging. Circ Res.

[120] Ungvari Z (2020). Mechanisms of vascular aging, A Geroscience Perspective: JACC Focus Seminar. J Am Coll Cardiol.

[121] Ho YH, Mendez-Ferrer S (2020). Microenvironmental contributions to hematopoietic stem cell aging. Haematologica.

[122] Schepers K, Campbell TB, Passegue E (2015). Normal and leukemic stem cell niches: insights and therapeutic opportunities. Cell Stem Cell.

[123] Maryanovich M (2018). Adrenergic nerve degeneration in bone marrow drives aging of the hematopoietic stem cell niche. Nat Med.

[124] ••Kusumbe AP, et al. Age-dependent modulation of vascular niches for haematopoietic stem cells. Nature. 2016;532(7599):380–4. **This study describes the prospect of restoring age-related vascular dysfunction to improve hematopoietic output.** 10.1038/nature17638 PMC5035541 27074508

[125] Poulos MG (2017). Endothelial transplantation rejuvenates aged hematopoietic stem cell function. J Clin Invest.

[126] Wang L (2014). Notch-dependent repression of miR-155 in the bone marrow niche regulates hematopoiesis in an NF-kappaB-dependent manner. Cell Stem Cell.

[127] Pober JS, Sessa WC (2007). Evolving functions of endothelial cells in inflammation. Nat Rev Immunol.

[128] Boettcher S (2014). Endothelial cells translate pathogen signals into G-CSF-driven emergency granulopoiesis. Blood.

[129] ••Ramalingam P, et al. Chronic activation of endothelial MAPK disrupts hematopoiesis via NFKB dependent inflammatory stress reversible by SCGF. Nat Commun. 2020;11(1):666. **This is the first study to experimentally describe that chronic vascular inflammation in the bone marrow is sufficient to drive premature HSC aging phenotypes.** 10.1038/s41467-020-14478-8 PMC6997369 32015345

[130] Young K, et al. Decline in IGF1 in the bone marrow microenvironment initiates hematopoietic stem cell aging. Cell Stem Cell. 2021;28(8):1473–1482 e7. 10.1016/j.stem.2021.03.017 PMC8349778 33848471

[131] Grunewald M, et al. Counteracting age-related VEGF signaling insufficiency promotes healthy aging and extends life span. Science. 2021;373(6554). 10.1126/science.abc8479 34326210

[132] Verovskaya EV, et al. Stromal inflammation is a targetable driver of hematopoietic aging. bioRxiv 2021. 2021;03(08):434485.

[133] Naveiras O (2009). Bone-marrow adipocytes as negative regulators of the haematopoietic microenvironment. Nature.

[134] Ambrosi TH, et al. Adipocyte accumulation in the bone marrow during obesity and aging impairs stem cell-based hematopoietic and bone regeneration. Cell Stem Cell. 2017;20(6):771–784 e6. 10.1016/j.stem.2017.02.009 PMC5459794 28330582

[135] Nishikawa K (2010). Maf promotes osteoblast differentiation in mice by mediating the age-related switch in mesenchymal cell differentiation. J Clin Invest.

[136] Connor KM (2018). Understanding metabolic changes in aging bone marrow. Exp Hematol Oncol.

[137] Kohli L, Passegue E (2014). Surviving change: the metabolic journey of hematopoietic stem cells. Trends Cell Biol.

[138] Tripathy D (2003). Elevation of free fatty acids induces inflammation and impairs vascular reactivity in healthy subjects. Diabetes.

